# Growth recovery and faltering through early adolescence in low- and middle-income countries: Determinants and implications for cognitive development

**DOI:** 10.1016/j.socscimed.2017.02.031

**Published:** 2017-04

**Authors:** Andreas Georgiadis, Liza Benny, Le Thuc Duc, Sheikh Galab, Prudhvikar Reddy, Tassew Woldehanna

**Affiliations:** a Brunel Business School, Brunel University London, Kingston Lane, Uxbridge, Middlesex, London, UB8 3PH, UK; b Department of International Development, University of Oxford, Oxford, UK; c Young Lives Study, University of Oxford, Oxford, UK; d Centre for Analysis and Forecasting, Vietnam Academy of Social Sciences, Hanoi, Viet Nam; e Centre for Economic and Social Studies (CESS), Hyderabad, India; f Department of Economics, Addis Ababa University, Ethiopia

**Keywords:** Child undernutrition, Post-infancy growth recovery and faltering, Growth trajectories, Cognitive development

## Abstract

Child chronic undernutrition, as measured by stunting, is prevalent in low- and middle-income countries and is among the major threats to child development. While stunting and its implications for cognitive development have been considered irreversible beyond early childhood there is a lack of consensus in the literature on this, as there is some evidence of recovery from stunting and that this recovery may be associated with improvements in cognition. Less is known however, about the drivers of growth recovery and the aspects of recovery linked to cognitive development. In this paper we investigate the factors associated with growth recovery and faltering through age 12 years and the implications of the incidence, timing, and persistence of post-infancy recovery from stunting for cognitive development using longitudinal data from Ethiopia, India, Peru, and Vietnam. We find that the factors most systematically associated with accelerated growth both before and after early childhood and across countries include mother's height, household living standards and shocks, community wages, food prices, and garbage collection. Our results suggest that post-infancy recovery from stunting is more likely to be systematically associated with higher achievement scores across countries when it is persistent and that associations between growth trajectories and cognitive achievement in middle childhood do not persist through early adolescence across countries. Overall, our findings indicate that growth after early childhood is responsive to changes in the household and community environments and that growth promotion after early childhood may yield improvements in child cognitive development.

## Highlights

• Living standards and costs are key predictors of child growth through age 12 years.
• Persistent post-infancy growth recovery is strongly associated with child cognition.
• Growth after early childhood is responsive to changes in the environment.
• Sustained post-infancy growth promotion may improve child cognition.

## 1 Introduction

Child undernutrition is one of the key risk factors to child survival, health, and development in low- and middle-income countries (LMICS) (Prendergast and Humphrey, 2014). The most common form of child undernutrition in LMICs is stunting, defined as height-for-age Z-score (HAZ) below −2, i.e. height that is more than two standard deviations below the median of the height distribution of a healthy-growing reference population of children of the same age and gender (WHO Multicentre Growth Reference Study Group, 2007). Although a number of studies have highlighted that stunting and its consequences for cognitive development are largely irreversible after early childhood (Victora et al., 2010), there is evidence both from the economics and the biomedical literature suggesting that growth recovery is possible beyond this period (Alderman et al., 2006, Prentice et al., 2013) and that it is positively associated with cognitive achievement (Crookston et al., 2013, Georgiadis et al., 2016).

Less is known however, about the factors associated with growth recovery and faltering at different periods following infancy. In particular, studies investigating predictors of growth recovery and faltering (Adair, 1999, Coly et al., 2006, Schott et al., 2013; see also Schott et al. (2013) for a survey of this literature) seem to explain a limited share of the variation in compensatory growth after early childhood, possibly because they consider a limited set of community predictors of catch-up growth. This seems to be an important gap in the literature, as aspects of the local environment such as standards of living and infrastructure have changed dramatically in recent years in low- and middle-income countries and are important policy levers linked to the reduction in stunting in several of these countries (Christiaensen and Alderman, 2004, Headey et al., 2016).

Moreover, studies considering the differences in cognitive achievement across children experiencing different post-infancy growth trajectories (Crookston et al., 2013, Fink and Rockers, 2014, Mendez and Adair, 1999; see also Georgiadis et al. (2016) for a survey of this literature) focus on the incidence of post-infancy growth recovery and ignore other aspects such as persistence and timing. Moreover, no study to our knowledge, to date, has investigated whether the associations between post-infancy growth recovery and cognitive achievement persist as children age.

In this paper, we address the aforementioned gaps in the literature using longitudinal data on children from Ethiopia, India, Peru, and Vietnam. In particular, we investigate a wide range of child, household, and community-level predictors of growth recovery and faltering at different periods from conception through early adolescence. A methodological innovation of our study is that we employ different estimators, including panel data estimators that deal with bias arising from fixed unobservables and a new measure of accelerated growth that addresses limitations of existing measures. We also examine whether the incidence, timing, and persistence of growth recovery, as measured by recovery from stunting, through middle childhood are significantly associated with cognitive achievement in this period and whether these associations persist through early adolescence.

## 2 Methods

### 2.1 Data

Our analysis uses data on around 8000 children born in 2001/2 in Ethiopia, India, Peru, and Vietnam (around 2000 in each country), collected as part of the Young Lives study (see Barnett et al. (2013) and Petrou and Kupek (2010) for details). The data include detailed information on a variety of indicators of children's health and development, such as height and cognitive achievement measures, and their household and community characteristics, when children were around 1, 5, 8, and 12 years old.

### 2.2 Measure of growth recovery and faltering

As a measure of growth recovery or faltering we use the change in child height relative to the change in height of the reference child measured in cm, as provided by the WHO standards (de Onis et al., 2007, WHO Multicentre Growth Reference Study Group., 2007), between two age points. This is a new measure that has many advantages over measures used by existing studies. For example, in contrast to the change in HAZ, it does not increase mechanically with age even if the height deficit relative to the reference, as measured in cm, remains the same or increases (Leroy et al., 2013, Lundeen et al., 2014) (see appendix for a detailed discussion).

### 2.3 Characterisation of growth trajectories

**Fig. 1:**
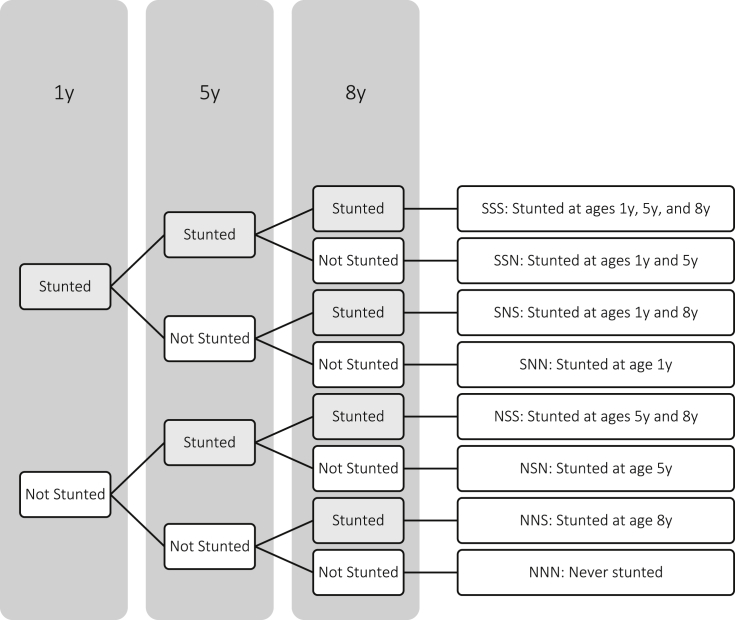
Characterisation of growth trajectories based on stunting status over three periods.

Child HAZ was calculated using child height and the 2006 WHO standard (WHO Multicentre Growth Reference Study Group, 2007) for children younger than 5 years and the 2007 WHO reference (de Onis et al., 2007) for children older than 5 years and an indicator for whether a child was stunted at each age was computed based on whether HAZ is less than −2 (WHO Multicentre Growth Reference Study Group, 2007). Child growth trajectories through age 8 years were characterised by stunting status at ages 1, 5, and 8 years that is an approach to modelling growth trajectories used in previous studies (Fink and Rockers, 2014). The different growth trajectories defined by this approach are presented in Fig. 1.

### 2.4 Measures of cognitive development

Cognitive development of children was assessed at ages 8 and 12 years using the Peabody Picture Vocabulary Test (PPVT), a widely-used test of receptive vocabulary, and a mathematics test at ages 8 and 12 years (Cueto and León, 2012). All tests were administered in different languages within each country to allow children to respond in the language they felt most comfortable. In our analysis, we used the number of correct answers in each test standardised by age in months as our measures of cognitive achievement.

### 2.5 Predictors of growth recovery and faltering

The identification of predictors of growth faltering and recovery at different ages was guided by the conceptual frameworks presented in Glewwe and Miguel (2007) and in Georgiadis (2017) who consider the determination of child health and cognitive development over different stages of the life course, and by previous empirical studies (Schott et al., 2013). Predictors included child characteristics, such as gender, birth order, age in months, and, only for growth between 8 and 12 years, whether the child has experienced puberty during this period; parental and household characteristics, such as caregiver's height, age at the index child's birth, years of schooling, and ethnicity (in the majority of cases the caregiver is the biological mother), father's years of schooling, household wealth index (see Woldehanna et al. (2011) for details of how the wealth index is constructed), and whether the household reported having been affected by shocks related to natural disasters, livelihood, and family events (see Table A.3 in the appendix for the type of shocks included in each category); and community characteristics, such as the number of credit-providing institutions in the community (i.e. banks, money lenders, etc.), that is used as a proxy of access to credit, price indices for food, medication, education, and other consumption items that are meant to capture aspects of the cost of living (see Table A.4 for details on the list of prices combined into each price index and how price indices were constructed), a wage index (see Table A.5 for details), a number of variables capturing different aspects of community's hygiene and health infrastructure (see Table A.5 for details), including whether water or air pollution is a problem in the community, whether there is access to improved water, improved sanitation, and to a hospital in the community, whether there is garbage disposal by truck, and finally the number of schools are used as a proxy of the learning environment in the community.

### 2.6 Predictors of cognitive development

Predictors of cognitive development other than growth trajectories were also identified using the conceptual frameworks of Glewwe and Miguel (2007) and Georgiadis (2017) as well as from previous empirical studies (Georgiadis et al., 2016). According to these frameworks, the predictors are a subset of those for growth faltering and recovery that excludes all factors that impact cognitive development through child growth trajectories such as mother's height, food and medication prices, and community hygiene and health infrastructure factors. Moreover, predictors of cognitive development also include household expenditure excluding expenditure on child health (Glewwe and Miguel, 2007).

### 2.7 Modelling and estimation

Specifications for growth faltering and recovery were estimated separately for four periods, conception to age 1 year, age 1–5 years, 5–8 years, and 8–12 years and for each country by ordinary least squares (OLS). Except for the period from conception to age 1 year, time-varying predictors were measured at the initial age. In the case of the period from conception to age 1 year, time-varying predictors were contemporaneous to the height-for-age measure, as no information on the values of these predictors at conception is available in the data. Nevertheless, contemporaneous values of these predictors are expected to be valid indicators of their values at conception. Moreover, the dependent variable in the period from conception to age 1 year was height-for-age in cm at age 1 year that, under the assumption that all children have the same height at conception, is equal to the change in height-for-age during this period. A specification for growth was also estimated using the longitudinal data for the periods between age 1 and 12 years by pooled OLS and first-differences. First-differences is preferred to fixed effects estimation because it relies on less strong assumptions regarding the exogeneity of the regressors (Cameron and Trivedi, 2005). OLS estimation allows us to estimate the coefficients of time-invariant regressors, whereas first-differences allows us to address bias in the estimated coefficients of explanatory variables arising from time-invariant unobservables.

The relationship between cognitive development and growth trajectories is modelled using 8 dummy variables or binary indicators, one for each growth trajectory presented in Fig. 1, each taking the value 1 if a given child exhibited the stunting history represented by the indicator, e.g. stunted at age 1 and 5 y (SSN), and is 0 otherwise. Separate specifications were estimated by OLS for each test score at age 8 and 12 years and for each country and all time-varying predictors were measured at age 8 years. All specifications also included controls for the language at which the test was administered and whether the test was administered at the child's native language. We also tested whether differences in achievement across children exhibiting different growth trajectories relative to the reference growth trajectory at age 8 years persist at age 12 years using a Chow test (Chow, 1960).

Children with implausible values of HAZ (absolute values of HAZ greater than 6) at any age were dropped from the analysis for relative growth (analysis on the relationship between growth trajectories and cognitive achievement did not drop children with implausible HAZ at age 12 years). In order to maximise the estimation sample, we imputed missing values of the control variables (prevalence 0.004%–2.8%) with their sample means (in the case of community variables we imputed using the sample mean in the same region and type of site (urban/rural)).

## 3 Results

### 3.1 Descriptive statistics

Descriptive statistics of the outcomes and child and household time-invariant characteristics used in our analysis are presented in Table 1 (see also Tables A.1 to A.5 for descriptive statistics of time-variant child, household, and community characteristics).

### 3.2 Determinants of accelerated child growth

Table 2 presents estimation results by OLS for each period of growth from conception to age 12 years. As there are four periods and many predictors, we identify as systematic predictors those that are significantly associated with relative growth in each period for at least two countries.

For the period between conception and age 1 year, accelerated growth is systematically associated with child gender and age, parental education, mother's height, household wealth, natural disaster and family shocks, prices of consumption goods, food items and medication, community wage, air pollution, and garbage collection by truck. For the period between age 1 and 5 years, patterns are similar as those identified for the period between conception and age 1 year with the difference that father's education, family shocks, food and medication prices are not significantly associated with child relative growth systematically across countries. Moreover, in contrast to relative growth through age 1 year, access to improved water and sanitation and child birth order significantly predict relative growth between age 1 and 5 years. The factors that systematically explain variation in child relative growth between 5 and 8 years include gender, birth order, and age, caregiver's height, household wealth, garbage collection by truck, and availability of hospital in the community. Finally, for the period between age 8 and 12 years the set of systematic predictors of growth is similar to that for the period between 5 and 8 years, but child birth order and hospital availability in the community are not significantly associated with child relative growth. In contrast to growth between age 5 and 8 years, differences in child relative growth between 8 and 12 years are explained by natural disaster shocks, prices of consumption goods and of food items, the average wage in the community, school availability, and the onset of puberty.

Table 3 presents estimation results with pooled OLS and first-differences using the panel sample from age 1–12 years for each country. Estimation results by OLS suggest that the most systematic time-invariant predictors of child relative growth in the period between 1 and 12 years include birth order, father's education, maternal height, and the number of credit providing institutions in the community when the child was age 1 year. Moreover, time-varying systematic predictors of child growth in this period, as identified by first-differences estimates, include child age, household wealth, natural disaster and family shocks, prices of food, medication, and education items, average wage, access to water and sanitation, and availability of hospital in the community.

### 3.3 Child growth trajectories and cognitive achievement

Table 4 presents OLS estimates of the associations between growth trajectories and achievement tests at age 8 and 12 years across countries (see Table A.6 in the appendix for full results). Results suggest that, relative to the reference group of children who were not stunted at any of the three age points, only children who were stunted at all ages have systematically lower achievement across countries and tests and over time.

Table 4 also presents results of F-tests of the equality of coefficients of each of the groups of children who were stunted at age 1 year and became non-stunted at some point later with those that remained stunted through age 8 years. Results suggest that children exhibited persistent recovery from stunting from 1 to 8 years (not stunted at age 5 and 8 years) performed better than children who remained stunted during this period in PPVT in R4 in Peru and Vietnam, in Maths in R3 and R4 in Ethiopia, and in Maths in R3 in India. Nevertheless, those who recovered temporarily from stunting between age 1 and 5 years perform similarly to those who have been stunted throughout this period, whereas children who recovered from stunting after age 5 years performed significantly better than those always stunted in both tests at age 8 years in Peru but not at age 12 years.

In the case of children who were not stunted at age 1 year, but became stunted either at age 5 or 8 years, those who became stunted at age 5 years and remained stunted through age 8 years exhibit systematically lower achievement than the reference at both ages in Ethiopia and Peru, in PPVT in India, and in Maths at age 8 years in Vietnam. Furthermore, those who became stunted at age 5 year, but recovered from stunting by age 8 years exhibit little difference in achievement scores relative to the reference, whereas children who became stunted at some point between 5 and 8 years have systematically lower scores than the reference mainly in PPVT.

Chow tests results suggest that, in some cases, as children age there are significant changes in achievement relative to the reference for some groups of children. In particular, we find that achievement improved between age 8 and 12 years, relative to the reference group, among children who were stunted at all three age points in Maths in India and Vietnam, and for those who became stunted at age 5 years and remained stunted through age 8 years in Vietnam, but deteriorated over time for those exhibited late recovery from stunting and temporary growth faltering in Peru.

## 4 Discussion

### 4.1 Drivers of growth recovery and faltering through early adolescence

Our analysis on the drivers of child growth recovery and faltering through early adolescence produces a number of key findings. First, we find that the child, household, and community factors considered as potential drivers of child growth at different periods seem to explain a considerably higher share of the variation in accelerated growth across children through age 5 years compared to the period from 5 to 12 years. Nevertheless, our results suggest that child growth after age 5 years and through early adolescence is also responsive to changes in the environment and that a number of factors are systematically associated with faster growth during this period. On the one hand, this evidence provides support to the current focus of growth-promoting interventions on children below the age of 5 years, but on the other hand suggests that growth failure through early childhood is not irreversible after this period and that there is potential for remediation of early growth deficits through interventions in later stages.

Similar to other studies (Adair, 1999, Schott et al., 2013), we find that maternal height, household living standards, parental education, child age, birth-order, and availability of hospital in the community are among the key predictors of accelerated growth in children. Nevertheless, a new finding arising from our analysis is that with the exception of mother's height, predetermined characteristics such as child birth-order and parental education do not predict child growth persistently across periods. This further suggests that the association of these factors with child growth is not expected to increase systematically with child age.

Our results also indicate that the direction of the association of community-level determinants of child growth at any given period is not the same across countries. These differences could be attributable to contextual differences across countries or to differences in the extent of confounding bias across countries arising from correlation with unobserved determinants of growth subsumed in the error term. Estimation of the specification in first-differences is expected to partly address the latter problem. Nevertheless, first-differences does not deal with time-varying confounders that cannot be ruled out and this is why we interpret our results as estimates of association rather than causal effects.

Our analysis identifies a new set of predictors of chid growth through age 12 years that include household shocks, particularly natural-disaster and family-related shocks, food prices, local wages, and the health and hygiene-related community infrastructure, such as garbage collection. Given the evidence that these factors are also systematically associated with the incidence of growth failure through infancy (Christiaensen and Alderman, 2004, Headey et al., 2016), our results highlight that interventions that aim to improve community economic conditions, hygiene, and health infrastructure may prevent growth failure in infants and young children and at the same time promote growth recovery in older children and adolescents.

### 4.2 Growth trajectories through middle childhood and cognitive achievement in middle childhood and early adolescence

Our results on the relationship between growth trajectories and cognitive achievement seem to be consistent with those from previous studies (Crookston et al., 2010, Crookston et al., 2013, Fink and Rockers, 2014) providing evidence that post-infancy growth recovery and faltering are significantly associated with cognitive achievement in childhood and adolescence. One key new finding, however, arising from our analysis is that this association is more marked in the case that post-infancy recovery and faltering are persistent.

There is also some evidence that the timing of growth recovery and faltering also matter for achievement. In particular, we find that children who recover from stunting after age 5 years have higher achievement scores at age 8 years than children who remained stunted in Peru and that children who became stunted after age 5 years have lower scores at age 12 years than children who were never stunted in Ethiopia and Peru. This could be partly explained in terms of biological mechanisms. In particular, according to the child development literature (Black et al., 1998, Thompson and Nelson, 2001), higher cognitive functions and other brain processes such as synaptogenesis, although they may peak at around age 1–2 years, continue to develop through adolescence and may be responsive to changes in child nutritional status after early childhood.

Another new finding of our study is that, although different growth trajectories through middle childhood are associated with significant differences in achievement at that stage, these differences do not necessarily persist through early adolescence across countries and that achievement can improve over time even among children who do not experience recovery from stunting. These changes over time could be explained by changes in the school and learning environment of children over time, that have been found to explain changes in cognitive function over time among children with high risk of stunting (Mendez and Adair, 1999, Sudfeld et al., 2015).

A limitation of our study, however, is that for some groups of children, such as the groups of children across countries who were stunted at age 1 and 8 years or stunted at age 8 years only, the small sample size makes it difficult to draw firm conclusions from the data on whether these groups are systematically different from the reference group in terms of cognitive achievement.

## 5 Conclusions

Although it has been suggested that child chronic undernutrition, as measured by child growth faltering, and the associated developmental setbacks are irreversible beyond early childhood, these hypotheses have not been supported uniformly by the evidence. Less is known, however, on the drivers of growth recovery and its implications for cognitive development. In this paper, we address these gaps in the literature using data on a cohort of children from Ethiopia, India, Peru, and Vietnam. Overall, our results suggest that child growth beyond early childhood is responsive to changes in the household and community environments and that sustained post-infancy growth promotion may be associated with improvements in child development.

## Figures and Tables

**Table 1:** Descriptive statistics of child outcomes and fixed child and household characteristics used in the analysis by country.

		Ethiopia	India	Peru	Vietnam
Height-for-age at 1 y (cm)		−3.75	−3.25	−3.22	−2.85
		(4.55)	(3.69)	(3.21)	(3.16)
Change in height-for-age between 1 y and 5 y (cm)		−2.92	−4.40	−3.89	−3.42
		(5.36)	(4.12)	(4.11)	(3.49)
Change in height-for-age between 5 y and 8 y (cm)		−0.01	−0.27	0.76	0.15
		(4.94)	(3.77)	(3.49)	(3.36)
Change in height-for-age between 8 y and 12 y (cm)		−4.30	−2.86	−1.64	−2.05
		(4.93)	(4.64)	(4.20)	(5.01)
Raw score in PPVT at age 8 y		21.23	27.58	29.33	37.00
		(11.84)	(21.13)	(17.67)	(18.13)
Raw score in Maths test at age 8 y		6.59	12.09	14.32	18.45
		(5.40)	(6.41)	(5.77)	(5.78)
Raw score in PPVT at age 12 y		38.46	43.12	85.83	58.35
		(8.73)	(7.86)	(17.55)	(8.23)
Raw score in Maths test at age 12 y		37.45	44.14	55.81	48.02
		(21.53)	(22.77)	(18.85)	(16.69)
SSS: Stunted at ages 1, 5, and 8 y	(%)	12	15	12	10
	(n)	214	276	217	185
SSN: Stunted at ages 1 and 5 y	(%)	7	4	5	4
	(n)	124	73	91	74
SNS: Stunted at ages 1 and 8 y	(%)	2	2	1	1
	(n)	36	37	18	19
SNN: Stunted at age 1 y	(%)	19	9	8	6
	(n)	339	166	145	111
NSS: Stunted at ages 5 and 8 y	(%)	4	9	5	6
	(n)	72	166	91	111
NSN: Stunted at age 5 y	(%)	7	7	10	4
	(n)	124	129	182	74
NNS: Stunted at age 8 y	(%)	3	3	2	3
	(n)	53	55	36	55
NNN: Never stunted	(%)	46	51	57	66
	(n)	820	938	1034	1220
Male		0.53	0.54	0.50	0.51
		(0.50)	(0.50)	(0.50)	(0.50)
First-born		0.23	0.39	0.37	0.46
		(0.42)	(0.49)	(0.48)	(0.50)
Second-born		0.20	0.39	0.26	0.36
		(0.40)	(0.49)	(0.44)	(0.48)
Third- or later-born		0.57	0.22	0.38	0.18
		(0.49)	(0.42)	(0.48)	(0.39)
Child experienced puberty by age 12 y		0.06	0.15	0.41	0.33
		(0.23)	(0.35)	(0.49)	(0.47)
Caregiver's schooling (years)		2.79	3.70	7.69	6.80
		(3.67)	(4.43)	(4.49)	(4.00)
Caregiver's height (cm)		158.66	151.47	149.98	152.18
		(5.92)	(5.98)	(5.37)	(5.88)
Caregiver's age at index child's birth (years)		28.21	23.01	27.01	27.79
		(8.88)	(5.43)	(8.07)	(8.56)
Father's schooling (years)		4.85	5.62	9.10	7.64
		(4.22)	(5.04)	(3.83)	(3.95)
Log household expenditure at age 8 y less spending on index child health at 5 and 8 y		4.82	6.68	5.19	6.00
		(0.59)	(0.56)	(0.59)	(0.61)
Number of observations		1782	1840	1814	1849

*Notes*: Statistics are means with standard deviations in parentheses, unless otherwise specified. Descriptive statistics for caregiver's ethnicity, community characteristics, language of administration in the tests, as well as for all time-varying child and household characteristics which were all also included in the analysis are reported in Tables A.1-A.5 in the appendix.

**Table 2:** OLS estimates of associations of child, household, and community characteristics with child growth from conception to 12 years.

	Ethiopia				India				Peru				Vietnam			
	Height-for-age at 1 y	Change in height-for-age between 1 & 5 y	Change in height-for-age between 5 & 8 y	Change in height-for-age between 8 & 12 y	Height-for-age at 1 y	Change in height-for-age between 1 & 5 y	Change in height-for-age between 5 & 8 y	Change in height-for-age between 8 & 12 y	Height-for-age at 1 y	Change in height-for-age between 1 & 5 y	Change in height-for-age between 5 & 8 y	Change in height-for-age between 8 & 12 y	Height-for-age at 1 y	Change in height-for-age between 1 & 5 y	Change in height-for-age between 5 & 8 y	Change in height-for-age between 8 & 12 y
Male	−0.67***	0.38	−0.40*	0.22	−0.18	0.02	0.06	0.05	−0.22*	0.66***	−0.31*	−0.29	−0.22*	0.51***	−0.46***	−0.22
	(0.19)	(0.23)	(0.23)	(0.23)	(0.16)	(0.18)	(0.17)	(0.22)	(0.13)	(0.17)	(0.17)	(0.19)	(0.13)	(0.14)	(0.15)	(0.22)
Second-born	0.16	−1.26***	0.69*	0.16	0.28	−0.29	−0.45**	0.14	0.10	−0.69***	−0.21	0.37	−0.08	−0.51***	−0.28	0.29
	(0.28)	(0.35)	(0.39)	(0.39)	(0.17)	(0.20)	(0.21)	(0.26)	(0.16)	(0.23)	(0.22)	(0.26)	(0.15)	(0.16)	(0.18)	(0.25)
Third- or later-born	0.09	−0.91***	0.11	0.42	−0.35	−0.60**	−0.63**	0.38	−0.08	−0.65***	−0.08	0.21	−0.01	−0.92***	0.03	−0.17
	(0.24)	(0.32)	(0.32)	(0.30)	(0.22)	(0.27)	(0.28)	(0.33)	(0.18)	(0.24)	(0.23)	(0.26)	(0.20)	(0.20)	(0.23)	(0.32)
Child age	−0.39***	0.30***	0.03	0.06**	−0.25***	0.26***	−0.02	−0.08***	−0.27***	0.27***	−0.05***	−0.06**	−0.19***	0.30***	−0.06**	0.01
	(0.03)	(0.03)	(0.03)	(0.03)	(0.02)	(0.03)	(0.02)	(0.03)	(0.02)	(0.02)	(0.02)	(0.03)	(0.02)	(0.02)	(0.02)	(0.03)
Puberty by 12 y				−0.66				2.46***				1.34***				1.64***
				(0.50)				(0.31)				(0.20)				(0.26)
Caregiver's height	0.08***	0.13***	0.01	0.02	0.12***	0.08***	0.04***	0.06***	0.13***	0.16***	0.04**	0.07***	0.11***	0.13***	0.03**	0.05***
	(0.02)	(0.02)	(0.02)	(0.02)	(0.02)	(0.02)	(0.02)	(0.02)	(0.01)	(0.02)	(0.02)	(0.02)	(0.01)	(0.01)	(0.01)	(0.02)
Caregiver's age at index child's birth	0.02	0.01	−0.00	−0.02	0.05***	−0.00	0.03	−0.07**	−0.01	0.02	0.01	−0.01	0.02	0.01	−0.01	0.02
	(0.01)	(0.02)	(0.01)	(0.01)	(0.02)	(0.02)	(0.02)	(0.03)	(0.01)	(0.01)	(0.01)	(0.01)	(0.01)	(0.01)	(0.01)	(0.01)
Caregiver's schooling	0.07**	0.00	−0.03	−0.01	0.07***	−0.01	0.07**	−0.02	0.08***	0.07***	0.01	−0.01	0.05**	0.07***	0.00	−0.03
	(0.03)	(0.04)	(0.04)	(0.05)	(0.02)	(0.03)	(0.03)	(0.03)	(0.02)	(0.03)	(0.03)	(0.03)	(0.02)	(0.03)	(0.03)	(0.04)
Father's schooling	0.05	0.06*	−0.04	−0.05	0.03	0.03	−0.03	0.05	0.05**	−0.00	0.04	0.05	0.05*	0.04	0.00	0.07*
	(0.03)	(0.04)	(0.03)	(0.04)	(0.02)	(0.02)	(0.02)	(0.03)	(0.02)	(0.03)	(0.03)	(0.03)	(0.03)	(0.03)	(0.03)	(0.04)
Household wealth index	4.03***	−0.77	2.83***	−0.98	1.22**	1.89***	0.72	0.54	1.60***	3.00***	0.27	2.03***	1.20**	1.87***	2.37***	1.86*
	(1.02)	(1.34)	(1.02)	(1.07)	(0.57)	(0.64)	(0.71)	(0.84)	(0.44)	(0.61)	(0.65)	(0.73)	(0.50)	(0.51)	(0.78)	(1.03)
Natural disaster shock	0.25	0.29	0.41	−0.70**	0.74***	−0.88***	0.18	0.37	0.14	−3.82**	−0.25	−0.50*	0.48**	−0.06	0.19	−0.18
	(0.31)	(0.36)	(0.31)	(0.32)	(0.26)	(0.28)	(0.21)	(0.31)	(0.39)	(1.92)	(0.21)	(0.29)	(0.23)	(0.26)	(0.21)	(0.33)
Livelihood shock	0.12	0.33	−0.08	−0.14	−0.15	−0.02	−0.12	−0.32	0.11	0.58***	−0.12	−0.06	0.06	−0.39*	0.02	−0.01
	(0.25)	(0.30)	(0.26)	(0.27)	(0.22)	(0.23)	(0.20)	(0.23)	(0.16)	(0.22)	(0.18)	(0.37)	(0.19)	(0.20)	(0.16)	(0.26)
Family shock	−0.56***	0.31	0.02	0.01	−0.52**	0.54**	0.22	0.03	−0.06	0.19	0.04	0.05	−0.12	0.03	−0.75***	−0.23
	(0.21)	(0.28)	(0.24)	(0.25)	(0.22)	(0.24)	(0.18)	(0.24)	(0.16)	(0.22)	(0.17)	(0.24)	(0.16)	(0.17)	(0.17)	(0.25)
Number of credit-providing institutions in community	0.27**	−0.13	−0.34**	0.25	0.09	0.08	0.21*	−0.13	0.10	0.09	0.12	0.11	0.09	−0.05	0.24**	0.16
	(0.11)	(0.13)	(0.14)	(0.30)	(0.09)	(0.11)	(0.12)	(0.14)	(0.06)	(0.08)	(0.08)	(0.08)	(0.07)	(0.08)	(0.10)	(0.11)
Consumption price index	−1.54***	0.91	0.38	2.30**	0.53	0.86**	−0.10	−2.02***	0.39	−0.27	2.19***	−0.20	−3.05*	4.35***	−0.49	−0.36
	(0.43)	(0.56)	(0.55)	(0.97)	(0.34)	(0.40)	(0.72)	(0.76)	(0.44)	(0.57)	(0.52)	(0.77)	(1.64)	(1.58)	(0.55)	(1.17)
Education price index	0.99	−0.67	1.75	0.01	0.40	−0.05	0.15	1.21***	−0.69	0.47	0.95	0.56	1.53**	0.46	0.17	3.22***
	(0.72)	(1.06)	(1.15)	(0.72)	(0.34)	(0.41)	(0.57)	(0.38)	(0.49)	(0.69)	(0.68)	(0.92)	(0.73)	(0.75)	(0.33)	(1.13)
Medicine price index	6.19**	−16.46***	0.87*	−1.48	0.34	−1.42**	0.13	−0.54	−0.81***	0.78**	0.18	0.41	0.11	0.70**	−0.21	−1.17**
	(3.02)	(4.17)	(0.50)	(1.38)	(0.45)	(0.56)	(0.58)	(0.61)	(0.28)	(0.38)	(0.29)	(0.41)	(0.28)	(0.31)	(0.27)	(0.58)
Food price index	4.05***	−4.25**	0.70	4.26*	4.54***	−5.50***	−0.68	2.44***	0.61*	−0.63	−1.33**	−0.16	1.50	−0.21	−0.60	1.22
	(1.25)	(1.68)	(0.80)	(2.19)	(0.79)	(0.92)	(1.05)	(0.90)	(0.35)	(0.49)	(0.54)	(0.38)	(1.17)	(1.21)	(0.60)	(1.04)
Community wage index	1.24*	−0.79	1.17	−1.30*	−2.49***	3.78***	−1.22**	0.48	−1.75***	0.49	−0.39	0.08	1.11**	0.80*	−0.54	−3.77***
	(0.68)	(0.88)	(0.96)	(0.71)	(0.59)	(0.61)	(0.54)	(0.74)	(0.52)	(0.70)	(0.38)	(0.57)	(0.44)	(0.45)	(0.76)	(1.30)
Community water pollution	0.67**	0.38	−0.20	0.38	0.33	−0.24	−0.24	0.49*	−0.28	−0.19	0.21	0.06	−0.24	−0.08	0.25	−0.52
	(0.31)	(0.40)	(0.34)	(0.40)	(0.22)	(0.26)	(0.27)	(0.30)	(0.20)	(0.26)	(0.18)	(0.23)	(0.17)	(0.18)	(0.21)	(0.33)
Community air pollution	−0.77	−0.09	−1.08*	−0.45	−1.03***	1.52***	0.10	−0.18	0.81***	−0.24	−0.02	−0.24	−0.19	−0.10	0.14	−0.02
	(0.55)	(0.87)	(0.58)	(0.45)	(0.32)	(0.41)	(0.20)	(0.30)	(0.23)	(0.31)	(0.18)	(0.22)	(0.20)	(0.20)	(0.27)	(0.46)
Improved water in community	0.47	1.03*	0.96	1.44*	−0.43	−0.29	0.10	0.22	−2.26***	0.02	−0.21	−0.36	−0.11	−0.73***	−1.06***	−0.79
	(0.42)	(0.55)	(1.41)	(0.77)	(0.34)	(0.38)	(0.81)	(0.28)	(0.87)	(0.87)	(0.44)	(0.49)	(0.23)	(0.23)	(0.27)	(0.89)
Improved sanitation in community	0.89***	−0.86**		0.30	0.33	−0.12	0.26	−0.96	−0.28	−0.62*	−0.08	−0.03	−0.15	0.87***		0.40
	(0.33)	(0.43)		(0.63)	(0.20)	(0.25)	(0.30)	(0.71)	(0.25)	(0.34)	(0.58)	(0.48)	(0.23)	(0.22)		(0.78)
Garbage disposal truck in community	−2.68***	1.00	−1.06*	1.02	−1.31***	1.91***	0.88**	−0.74*	0.37*	0.22	−0.07	−0.21	0.41	1.09***	0.51*	1.33***
	(0.66)	(0.88)	(0.60)	(0.73)	(0.30)	(0.37)	(0.42)	(0.42)	(0.21)	(0.29)	(0.33)	(0.36)	(0.27)	(0.32)	(0.29)	(0.33)
Hospital in community	−2.41***	2.07***	−0.93**	−1.04	−0.13	0.12	−0.12	−0.17	0.21	0.25	0.43*	0.24	−0.15	−1.14*	−1.74***	−0.58
	(0.36)	(0.53)	(0.44)	(0.73)	(0.19)	(0.21)	(0.22)	(0.33)	(0.18)	(0.24)	(0.26)	(0.28)	(0.66)	(0.64)	(0.61)	(0.73)
Number of schools in community			0.62	−0.04			0.04	0.11***			0.10	0.22			0.19	−0.18***
			(0.42)	(0.13)			(0.11)	(0.04)			(0.15)	(0.16)			(0.15)	(0.07)

R-squared	0.237	0.183	0.0633	0.0625	0.209	0.172	0.0655	0.0656	0.321	0.240	0.0455	0.0772	0.235	0.303	0.0627	0.107
Observations	1782	1782	1782	1782	1840	1840	1840	1840	1814	1814	1814	1814	1849	1849	1849	1849

*Notes*: Robust standard errors are in parentheses. *** significant at 1%, ** significant at 5%, * significant at 10%. Regressions include controls for caregiver's ethnicity, but estimates are not reported. Time-varying controls in specifications of change in height are from the initial period and from the same period in specifications for height-for-age at age 1 year.

**Table 3:** Pooled OLS and first-differences estimates of associations of child, household, and community characteristics with the child growth based on longitudinal data from periods during which children across countries were between 1 to 5, 5 to 8, and 8 to 12 Years.

	Ethiopia		India		Peru		Vietnam	
	Change in height-for-age		Change in height-for-age		Change in height-for-age		Change in height-for-age	
	Pooled OLS	First differences	Pooled OLS	First differences	Pooled OLS	First differences	Pooled OLS	First differences
Male	0.04		−0.04		0.11		−0.03	
	(0.10)		(0.09)		(0.09)		(0.09)	
Second-born	−0.13		−0.21**		−0.23*		−0.14	
	(0.15)		(0.11)		(0.12)		(0.11)	
Third- or later-born	−0.20		−0.34**		−0.21*		−0.43***	
	(0.13)		(0.14)		(0.12)		(0.13)	
Child age	0.13***	0.78***	0.05***	0.20**	0.02*	−0.22***	0.06***	0.01
	(0.01)	(0.10)	(0.01)	(0.09)	(0.01)	(0.04)	(0.01)	(0.12)
Caregiver's height	0.05***		0.06***		0.09***		0.08***	
	(0.01)		(0.01)		(0.01)		(0.01)	
Caregiver's age at index child's birth	−0.00		−0.01		0.01		0.01	
	(0.01)		(0.01)		(0.01)		(0.01)	
Caregiver's schooling	−0.03		0.02		0.03		−0.00	
	(0.02)		(0.02)		(0.02)		(0.02)	
Father's schooling	−0.02		0.02		0.03*		0.03*	
	(0.02)		(0.01)		(0.02)		(0.02)	
Household wealth index	1.06**	2.52**	1.16***	−1.39	2.01***	0.85	2.05***	2.44***
	(0.51)	(1.20)	(0.40)	(0.95)	(0.34)	(0.82)	(0.36)	(0.86)
Natural disaster shock	−0.31*	−0.47	−0.30**	0.05	−0.24	−0.61**	−0.08	−0.07
	(0.18)	(0.32)	(0.14)	(0.26)	(0.18)	(0.25)	(0.16)	(0.23)
Livelihood shock	−0.06	0.22	−0.29**	−0.21	0.10	0.29	−0.19	0.01
	(0.15)	(0.24)	(0.13)	(0.21)	(0.14)	(0.21)	(0.13)	(0.19)
Family shock	0.21	0.35	0.22	0.57***	0.14	0.26	−0.30**	−0.46**
	(0.15)	(0.23)	(0.13)	(0.20)	(0.12)	(0.18)	(0.12)	(0.18)
Number of credit-providing institutions in the community	−0.09		0.07		0.13***		0.16***	
	(0.06)		(0.06)		(0.04)		(0.05)	
Consumption price index	0.75***	−0.08	0.28	0.22	0.35	1.15**	0.44	0.40
	(0.21)	(0.46)	(0.28)	(0.43)	(0.33)	(0.52)	(0.37)	(0.47)
Education price index	−0.00	−0.01	0.67***	0.74**	1.01***	0.92*	1.24***	1.28***
	(0.21)	(0.30)	(0.18)	(0.30)	(0.38)	(0.52)	(0.23)	(0.32)
Medicine price index	0.03	0.26	−0.62**	−2.65***	0.46**	0.49*	−0.15	0.19
	(0.29)	(0.41)	(0.28)	(0.49)	(0.18)	(0.29)	(0.15)	(0.26)
Food price index	−0.99**	−2.40***	−0.89*	−0.41	−0.62**	−1.31**	−0.57	−1.80**
	(0.38)	(0.62)	(0.47)	(0.76)	(0.25)	(0.51)	(0.42)	(0.77)
Community wage index	0.44	−0.85	0.22	−0.73	−1.03***	−0.93*	−0.22	−1.48**
	(0.30)	(0.55)	(0.34)	(0.64)	(0.27)	(0.48)	(0.34)	(0.58)
Community water pollution	0.55***	0.70**	0.05	−0.34	−0.25**	−0.28	−0.01	0.12
	(0.16)	(0.33)	(0.15)	(0.24)	(0.11)	(0.17)	(0.12)	(0.17)
Community air pollution	−0.11	−0.64**	0.16	−0.00	−0.21*	−0.14	0.19	0.27
	(0.19)	(0.31)	(0.14)	(0.21)	(0.12)	(0.18)	(0.13)	(0.21)
Improved water in community	1.01***	2.27***	−0.29	0.21	−0.30	0.26	−0.79***	−1.16***
	(0.23)	(0.35)	(0.22)	(0.36)	(0.28)	(0.46)	(0.16)	(0.23)
Improved sanitation in community	−0.23	−1.45***	0.10	0.08	−0.17	−0.56	1.38***	1.85***
	(0.25)	(0.47)	(0.17)	(0.25)	(0.26)	(0.43)	(0.21)	(0.34)
Garbage disposal truck in community	0.58**	−0.71	0.57***	−0.22	0.10	0.26	1.00***	1.32***
	(0.28)	(0.62)	(0.19)	(0.41)	(0.18)	(0.37)	(0.16)	(0.32)
Hospital in community	0.25	0.42	−0.10	−0.55*	0.28**	0.27	−1.27***	−2.06***
	(0.18)	(0.32)	(0.13)	(0.32)	(0.14)	(0.32)	(0.36)	(0.68)
R-squared	0.139	0.204	0.172	0.232	0.246	0.289	0.198	0.227
Observations	5346	3564	5520	3680	5442	3628	5547	3698

*Notes*: Standard errors clustered at the child level in parentheses. *** significant at 1%, ** significant at 5%, * significant at 10%. Regressions include controls for caregiver's ethnicity and period dummies, but estimates are not reported. Time-varying controls in all specifications are from the initial period.

**Table 4:** OLS estimates of associations of child growth trajectories from age 1 to 8 years with achievement scores at ages 8 and 12 years.

	Ethiopia				India				Peru				Vietnam			
	PPVT at 8 y	Maths at 8 y	PPVT at 12 y	Maths at 12 y	PPVT at 8 y	Maths at 8 y	PPVT at 12 y	Maths at 12 y	PPVT at 8 y	Maths at 8 y	PPVT at 12 y	Maths at 12 y	PPVT at 8 y	Maths at 8 y	PPVT at 12 y	Maths at 12 y
SSS: Stunted at ages 1, 5, and 8 y	−0.19***	−0.30***	−0.23***	−0.29***	−0.31***	−0.42***	−0.36***	−0.23***	−0.23***	−0.32***	−0.32***	−0.23***	−0.12*	−0.34***	−0.25***	−0.17**
	(0.06)	(0.07)	(0.07)	(0.07)	(0.06)	(0.06)	(0.07)	(0.06)	(0.07)	(0.07)	(0.06)	(0.07)	(0.07)	(0.07)	(0.08)	(0.07)
SSN: Stunted at ages 1 and 5 y	−0.13	−0.16*	−0.07	−0.13	−0.24**	−0.26**	−0.31***	−0.17	−0.03	−0.11	−0.30***	−0.12	−0.14	−0.16	−0.15	−0.14
	(0.08)	(0.09)	(0.08)	(0.09)	(0.09)	(0.11)	(0.11)	(0.11)	(0.10)	(0.10)	(0.10)	(0.10)	(0.08)	(0.10)	(0.13)	(0.10)
SNS: Stunted at ages 1 and 8 y	−0.41***	−0.23	−0.48**	−0.10	−0.35***	−0.47***	−0.19	−0.28	−0.57***	−0.63***	−0.48***	−0.52**	−0.36*	−0.55**	−0.46	−0.39**
	(0.12)	(0.16)	(0.19)	(0.17)	(0.13)	(0.15)	(0.16)	(0.18)	(0.20)	(0.23)	(0.17)	(0.26)	(0.19)	(0.26)	(0.31)	(0.16)
SNN: Stunted at age 1 y	−0.11**	−0.16***	−0.11**	−0.12*	−0.21***	−0.21***	−0.26***	−0.19**	−0.15**	−0.13*	−0.14**	−0.11	−0.02	−0.15	0.02	−0.11
	(0.05)	(0.06)	(0.05)	(0.06)	(0.08)	(0.07)	(0.07)	(0.07)	(0.07)	(0.08)	(0.07)	(0.08)	(0.10)	(0.09)	(0.10)	(0.10)
NSS: Stunted at ages 5 and 8 y	−0.26**	−0.41***	−0.41***	−0.25*	−0.16**	−0.03	−0.15**	−0.12	−0.29***	−0.26**	−0.36***	−0.25**	−0.10	0.17**	−0.09	−0.06
	(0.11)	(0.11)	(0.11)	(0.14)	(0.07)	(0.08)	(0.08)	(0.08)	(0.09)	(0.10)	(0.09)	(0.11)	(0.10)	(0.08)	(0.10)	(0.09)
NSN: Stunted at age 5 y	−0.03	−0.08	−0.02	−0.05	0.18*	0.02	0.00	−0.06	−0.10	−0.09	−0.23***	−0.14*	0.01	0.05	−0.10	0.15
	(0.09)	(0.08)	(0.08)	(0.09)	(0.11)	(0.10)	(0.08)	(0.08)	(0.06)	(0.07)	(0.06)	(0.08)	(0.09)	(0.09)	(0.11)	(0.11)
NNS: Stunted at age 8 y	−0.15	−0.23	−0.29*	−0.29*	0.07	0.21	−0.05	0.07	−0.23**	−0.07	−0.33***	−0.13	−0.28***	0.14	−0.18	−0.05
	(0.14)	(0.16)	(0.16)	(0.16)	(0.14)	(0.15)	(0.11)	(0.14)	(0.11)	(0.15)	(0.13)	(0.14)	(0.11)	(0.13)	(0.14)	(0.13)
Constant	0.38	−3.82*	1.28	−2.94	2.69*	0.43	−0.75	−0.45	−5.49***	−4.68**	−3.65**	−0.84	−1.74	−2.78**	−2.47*	−4.91***
	(1.91)	(2.04)	(1.88)	(2.37)	(1.63)	(1.49)	(1.55)	(1.42)	(1.69)	(2.32)	(1.65)	(2.19)	(1.35)	(1.24)	(1.42)	(1.27)
R-squared	0.41	0.36	0.41	0.28	0.16	0.22	0.23	0.25	0.44	0.31	0.40	0.26	0.27	0.29	0.28	0.23
Observations	1540	1501	1540	1501	1813	1784	1813	1784	1715	1756	1715	1756	1737	1778	1737	1778
**P-values of tests of equality of coefficients**																
*F-tests of equality of coefficients between groups of children who recovered from stunting and those who remained stunted*																
SSS = SNN	0.22	0.07	0.12	0.05	0.26	0.02	0.25	0.59	0.32	0.05	0.03	0.21	0.35	0.10	0.02	0.66
SSS = SNS	0.10	0.69	0.22	0.29	0.76	0.74	0.32	0.80	0.10	0.19	0.38	0.27	0.21	0.41	0.52	0.18
SSS = SSN	0.44	0.17	0.10	0.15	0.49	0.18	0.65	0.61	0.08	0.05	0.78	0.32	0.86	0.12	0.48	0.80
*Chow tests of equality of coefficients at ages 8 and 12 y*																
SSS			0.62	0.87			0.47	0			0.20	0.18			0.17	0.03
SSN			0.48	0.73			0.54	0.41			0	0.93			0.92	0.88
SNS			0.68	0.42			0.45	0.29			0.56	0.53			0.72	0.59
SNN			0.90	0.53			0.59	0.79			0.91	0.72			0.75	0.72
NSS			0.27	0.33			0.97	0.24			0.38	0.94			0.92	0.02
NSN			0.97	0.70			0.11	0.36			0.03	0.51			0.43	0.41
NNS			0.28	0.73			0.51	0.32			0.55	0.72			0.46	0.24

*Notes*: Robust standard errors in parentheses. *** significant at 1%, ** significant at 5%, * significant at 10%. Dependent variables are age-normalised test scores. Regressions include controls for child gender, birth order, age in months in 2002, time elapsed between interviews at age 1 and 5 years and between interviews at age 5 and 8 years, caregiver's age at childbirth, ethnicity, and schooling, father's schooling, the natural logarithm of household monthly per capita expenditure at age 8 years excluding expenditure on child health at age 5 and 8 years, number of schools, wage index, education price index, and consumption price index in the community at age 8 years, and number of credit providing institutions in the community at age 1 year, and the language of administration of the tests, and whether the tests were administered in the child's native tongue. Full estimation results are reported in Table A.6 in the Appendix.
