# Supplementary material for: Growth recovery and faltering through early adolescence in low- and middle-income countries: Determinants and implications for cognitive development
Source: Soc Sci Med. 2017 Apr;179:81–90. doi: 10.1016/j.socscimed.2017.02.031 (PMC5380196; doi:10.1016/j.socscimed.2017.02.031)
Supplement: Supplementary Material [file mmc1.docx]

**APPENDIX**

**Advantages of the Measure of Child Growth Adopted Relative to Other Measures in the Literature**

There is no universally agreed definition of growth recovery or catch-up growth (Adair, 1999) and this is why a range of different measures have been employed across studies that have different limitations and advantages and are expected to lead to different conclusions (Cameron, Preece, & Cole, 2005; Eckhardt, Gordon-Larsen, & Adair, 2005; Godoy et al., 2010). Two commonly used measures in the literature have been the change in child’s height-for-age Z-score (HAZ) (Eckhardt et al., 2005) and the change in height in cm (Adair, 1999) between two points in time. The key limitation of the latter measure, as highlighted in the literature, is that it does not use a reference measure or benchmark relative to which the faster or slower rate of growth during a given stage of life could be assessed (Adair, 1999). A recently highlighted limitation of the change in HAZ measure is that it may increase mechanically in a population with a higher share of children initially below the reference, as the standard deviation of WHO reference height distribution increases with age, even if the height deficit in these children relative to the reference, as measured in cm, remains the same or increases (Leroy, Ruel, & Habicht, 2013; Lundeen et al., 2014). In terms of modelling the determinants of child growth recovery this is expected to lead to inefficient estimation, as it increases the share of variation in the measure of child growth that cannot be explained by observed predictors. Moreover, other limitations of the change in HAZ that have not been highlighted before are that the unit of measurement of child growth relative to the reference is neither clear nor correct.

In order to see this, consider the following equation expressing the change in HAZ of child $i$ between period $t$ and $t-1$:

$${\Delta HAZ}_{it}=\frac{H_{it}-H_{rt}}{\sigma_{rt}}-\frac{H_{it-1}-H_{rt-1}}{\sigma_{rt-1}} \left( 1 \right)$$

where $H_{it}$, $H_{it-1}$ is the height of child $i$ measured in cm in periods $t$ and $t-1$ respectively and $H_{rt}$, $H_{rt-1}$ is the height of the reference child measured in cm in periods $t$ and $t-1$ respectively, and $\sigma_{rt}$, $\sigma_{rt-1}$ are the standard deviations of the reference height distributions in periods $t$ and $t-1$ respectively. As suggested by equation (1), because $\sigma_{rt}\neq\sigma_{rt-1}$, it is not clear what is the unit of measurement of child growth relative to the reference child between $t$ and $t-1$. Moreover, equation (1) indicates that the numerators in (1) measure child growth relative to the growth of the reference between two points in time, whereas the standard deviations in the denominators are those of the height distribution at a given point in time. Therefore, unless $\frac{H_{it-1}-H_{rt-1}}{\sigma_{rt-1}}$=0, the standard deviations of the reference height distribution do not provide the correct unit of measurement for the change in height of a given child relative to the change in height of the reference child in a given period.

Using as a measure of growth recovery and faltering the change in height of a given child relative to the change in height in the reference child measured in cm between two different ages addresses the aforementioned limitations of the two commonly used measures. Strictly speaking this is a measure of accelerated or decelerated child growth, relative to the reference, during a given period and not a measure of growth recovery or faltering, as the latter two require that children initially exhibit growth failure or normal growth respectively (Adair, 1999). Nevertheless, accelerated and decelerated growth are necessary conditions of growth recovery and faltering respectively.

**Table A.1: Descriptive Statistics of Caregiver’s Ethnicity by Country**

|  | Ethiopia | India | Peru | Vietnam |
| --- | --- | --- | --- | --- |
| Amhara | 0.29 |  |  |  |
|  | (0.45) |  |  |  |
| Gurage | 0.08 |  |  |  |
|  | (0.27) |  |  |  |
| Hadiya | 0.05 |  |  |  |
|  | (0.21) |  |  |  |
| Oromo | 0.21 |  |  |  |
|  | (0.41) |  |  |  |
| Sidama | 0.05 |  |  |  |
|  | (0.23) |  |  |  |
| Tigrian | 0.22 |  |  |  |
|  | (0.41) |  |  |  |
| Wolayta | 0.06 |  |  |  |
|  | (0.24) |  |  |  |
| Scheduled castes |  | 0.18 |  |  |
|  |  | (0.38) |  |  |
| Scheduled tribes |  | 0.13 |  |  |
|  |  | (0.34) |  |  |
| Other backward classes |  | 0.48 |  |  |
|  |  | (0.50) |  |  |
| Quechua |  |  | 0.19 | 0.00 |
|  |  |  | (0.40) | (0.00) |
| White |  |  | 0.03 |  |
|  |  |  | (0.18) |  |
| Mixed ethnicity |  |  | 0.72 |  |
|  |  |  | (0.45) |  |
| Kinh |  |  |  | 0.86 |
|  |  |  |  | (0.34) |
| H’mong |  |  |  | 0.05 |
|  |  |  |  | (0.22) |
| Other ethnicities | 0.04 | 0.21 | 0.05 | 0.09 |
|  | (0.20) | (0.41) | (0.21) | (0.28) |
| Number of observations | 1782 | 1840 | 1814 | 1849 |

*Notes*: Statistics are means with standard deviations in parentheses.

**Table A.2: Descriptive Statistics of Language of Administration of Tests by Country**

|  | Ethiopia | India | Peru | Vietnam |
| --- | --- | --- | --- | --- |
| *Child’s native language* |  |  |  |  |
| Amharic | 0.43 |  |  |  |
|  | (0.50) |  |  |  |
| Oromiya | 0.21 |  |  |  |
|  | (0.41) |  |  |  |
| Tigrigna | 0.20 |  |  |  |
|  | (0.40) |  |  |  |
| Other Ethiopian language | 0.16 |  |  |  |
|  | (0.37) |  |  |  |
| Telugu |  | 0.92 |  |  |
|  |  | (0.27) |  |  |
| Kannada |  | 0.03 |  |  |
|  |  | (0.18) |  |  |
| Other Indian language |  | 0.04 |  |  |
|  |  | (0.20) |  |  |
| Spanish |  |  | 0.89 |  |
|  |  |  | (0.31) |  |
| Quechua |  |  | 0.11 |  |
|  |  |  | (0.31) |  |
| Other |  |  | 0.00 |  |
|  |  |  | (0.02) |  |
| Vietnamese |  |  |  | 0.80 |
|  |  |  |  | (0.40) |
| H'mong |  |  |  | 0.04 |
|  |  |  |  | (0.20) |
| Other Vietnamese language |  |  |  | 0.16 |
|  |  |  |  | (0.36) |
| *Language of administration of cognitive tests* |  |  |  |  |
| PPVT at age 8 y administered in native language | 0.99 | 0.81 | 0.91 | 0.81 |
|  | (0.07) | (0.39) | (0.28) | (0.39) |
| PPVT at age 12 y administered in native language | 0.98 | 0.92 | 0.98 | 0.92 |
|  | (0.15) | (0.27) | (0.12) | (0.28) |
| Maths test at age 8 y administered in native language | 0.99 | 0.75 | 0.91 | 0.82 |
|  | (0.11) | (0.43) | (0.29) | (0.39) |
| Maths test at age 12 y administered in native language | 1.00 | 0.92 | 0.98 | 0.92 |
|  | (0.00) | (0.27) | (0.12) | (0.28) |
| Number of observations | 1688 | 1830 | 1765 | 1828 |

*Notes*: Statistics are means with standard deviations in parentheses.

**Table A.3: Descriptive Statistics of Time-varying Child and Household Characteristics by Country**

|  | 2002 | | | | 2006 | | | | 2009 | | | |
| --- | --- | --- | --- | --- | --- | --- | --- | --- | --- | --- | --- | --- |
|  | Ethiopia | India | Peru | Vietnam | Ethiopia | India | Peru | Vietnam | Ethiopia | India | Peru | Vietnam |
| Child age (months) | 11.65 | 11.76 | 11.57 | 11.68 | 61.82 | 64.27 | 63.51 | 63.14 | 97.44 | 95.33 | 94.93 | 96.57 |
|  | (3.55) | (3.46) | (3.53) | (3.16) | (3.81) | (3.84) | (4.67) | (3.59) | (3.92) | (3.81) | (3.60) | (3.71) |
| Household wealth index | 0.21 | 0.41 | 0.43 | 0.43 | 0.28 | 0.46 | 0.47 | 0.49 | 0.33 | 0.52 | 0.54 | 0.61 |
|  | (0.17) | (0.20) | (0.24) | (0.21) | (0.18) | (0.20) | (0.23) | (0.18) | (0.18) | (0.18) | (0.21) | (0.18) |
| *Household experienced a natural disaster shock* |  |  |  |  |  |  |  |  |  |  |  |  |
| Natural disaster shock | 0.22 | 0.23 | 0.00 | 0.09 |  |  |  |  |  |  |  |  |
|  | (0.42) | (0.42) | (0.03) | (0.29) |  |  |  |  |  |  |  |  |
| Drought | 0.29 | 0.28 | 0.07 | 0.07 | 0.29 | 0.28 | 0.07 | 0.07 | 0.29 | 0.28 | 0.07 | 0.07 |
|  | (0.45) | (0.45) | (0.26) | (0.25) | (0.45) | (0.45) | (0.26) | (0.25) | (0.45) | (0.45) | (0.26) | (0.25) |
| Too much rain or flooding | 0.14 | 0.06 | 0.04 | 0.10 | 0.14 | 0.06 | 0.04 | 0.10 | 0.14 | 0.06 | 0.04 | 0.10 |
|  | (0.35) | (0.24) | (0.20) | (0.31) | (0.35) | (0.24) | (0.20) | (0.31) | (0.35) | (0.24) | (0.20) | (0.31) |
| Erosion | 0.08 | 0.00 | 0.00 | 0.02 | 0.08 | 0.00 | 0.00 | 0.02 | 0.08 | 0.00 | 0.00 | 0.02 |
|  | (0.27) | (0.00) | (0.07) | (0.14) | (0.27) | (0.00) | (0.07) | (0.14) | (0.27) | (0.00) | (0.07) | (0.14) |
| Frost or hailstorms | 0.11 | 0.00 | 0.12 | 0.03 | 0.11 | 0.00 | 0.12 | 0.03 | 0.11 | 0.00 | 0.12 | 0.03 |
|  | (0.31) | (0.05) | (0.32) | (0.16) | (0.31) | (0.05) | (0.32) | (0.16) | (0.31) | (0.05) | (0.32) | (0.16) |
| *Household experienced a livelihood shock* |  |  |  |  |  |  |  |  |  |  |  |  |
| Livestock death | 0.20 | 0.06 | 0.01 | 0.04 | 0.26 | 0.06 | 0.01 | 0.11 | 0.32 | 0.13 | 0.02 | 0.10 |
|  | (0.40) | (0.24) | (0.10) | (0.19) | (0.44) | (0.24) | (0.11) | (0.32) | (0.47) | (0.34) | (0.13) | (0.30) |
| Pests or diseases affecting livestock |  |  |  |  | 0.04 | 0.01 | 0.04 | 0.09 | 0.11 | 0.04 | 0.04 | 0.06 |
|  |  |  |  |  | (0.20) | (0.10) | (0.19) | (0.29) | (0.31) | (0.21) | (0.20) | (0.24) |
| Crop failure | 0.40 | 0.29 | 0.02 | 0.10 | 0.21 | 0.13 | 0.03 | 0.13 | 0.26 | 0.32 | 0.03 | 0.18 |
|  | (0.49) | (0.45) | (0.14) | (0.31) | (0.41) | (0.34) | (0.16) | (0.33) | (0.44) | (0.47) | (0.18) | (0.38) |
| Pests or diseases affecting crops before harvesting |  |  |  |  | 0.07 | 0.07 | 0.04 | 0.09 | 0.07 | 0.14 | 0.09 | 0.17 |
|  |  |  |  |  | (0.26) | (0.26) | (0.19) | (0.28) | (0.25) | (0.35) | (0.28) | (0.37) |
| Pests or diseases leading to storage losses |  |  |  |  | 0.03 | 0.00 | 0.00 | 0.03 | 0.03 | 0.01 | 0.01 | 0.02 |
|  |  |  |  |  | (0.16) | (0.06) | (0.05) | (0.16) | (0.16) | (0.11) | (0.08) | (0.13) |
| Theft of livestock | 0.02 | 0.01 | 0.00 | 0.01 | 0.04 | 0.01 | 0.02 | 0.05 |  |  | 0.02 |  |
|  | (0.14) | (0.10) | (0.07) | (0.08) | (0.19) | (0.10) | (0.13) | (0.22) |  |  | (0.15) |  |
| Theft of crops | 0.03 | 0.03 | 0.00 | 0.00 | 0.05 | 0.01 | 0.01 | 0.01 |  |  | 0.01 |  |
|  | (0.18) | (0.17) | (0.02) | (0.03) | (0.22) | (0.08) | (0.11) | (0.08) |  |  | (0.11) |  |
| Job loss | 0.12 | 0.05 | 0.15 | 0.05 | 0.10 | 0.01 | 0.03 | 0.03 | 0.12 | 0.02 |  | 0.06 |
|  | (0.33) | (0.22) | (0.35) | (0.22) | (0.30) | (0.10) | (0.17) | (0.16) | (0.32) | (0.12) |  | (0.24) |
| Destruction or closure of place of employment |  |  |  |  | 0.01 | 0.00 | 0.04 | 0.00 |  |  | 0.03 |  |
|  |  |  |  |  | (0.09) | (0.06) | (0.19) | (0.02) |  |  | (0.17) |  |
| Confiscation of assets |  |  |  |  | 0.01 | 0.00 |  | 0.00 |  |  |  |  |
|  |  |  |  |  | (0.08) | (0.06) |  | (0.00) |  |  |  |  |
| Crime | 0.02 | 0.03 | 0.01 | 0.00 |  |  |  |  |  |  |  |  |
|  | (0.15) | (0.17) | (0.08) | (0.02) |  |  |  |  |  |  |  |  |
| Theft of tools for production |  |  |  |  | 0.02 | 0.01 | 0.01 | 0.01 | 0.10 | 0.05 | 0.02 | 0.05 |
|  |  |  |  |  | (0.14) | (0.10) | (0.11) | (0.08) | (0.30) | (0.21) | (0.14) | (0.23) |
| Theft of cash |  |  |  |  | 0.05 | 0.02 | 0.06 | 0.01 |  |  | 0.06 |  |
|  |  |  |  |  | (0.21) | (0.13) | (0.23) | (0.10) |  |  | (0.24) |  |
| Theft of housing or consumer goods |  |  |  |  | 0.02 | 0.02 | 0.07 | 0.03 |  |  | 0.06 |  |
|  |  |  |  |  | (0.14) | (0.13) | (0.25) | (0.17) |  |  | (0.24) |  |
| Crime resulting in death or disablement of adult household members |  |  |  |  | 0.00 | 0.00 | 0.00 | 0.00 |  |  | 0.00 |  |
|  |  |  |  |  | (0.07) | (0.02) | (0.02) | (0.07) |  |  | (0.03) |  |
| Household experienced a family shock | 0.31 | 0.22 | 0.20 | 0.21 | 0.58 | 0.45 | 0.33 | 0.45 | 0.52 | 0.27 | 0.22 | 0.33 |
|  | (0.46) | (0.42) | (0.40) | (0.41) | (0.49) | (0.50) | (0.47) | (0.50) | (0.50) | (0.45) | (0.42) | (0.47) |
| Illness of household member | 0.13 | 0.17 | 0.09 | 0.14 |  |  |  |  |  |  |  |  |
|  | (0.34) | (0.37) | (0.29) | (0.35) |  |  |  |  |  |  |  |  |
| Illness of index child's father |  |  |  |  | 0.16 | 0.08 | 0.04 | 0.08 | 0.16 | 0.07 | 0.03 | 0.10 |
|  |  |  |  |  | (0.37) | (0.27) | (0.19) | (0.26) | (0.37) | (0.26) | (0.17) | (0.31) |
| Illness of index child's mother |  |  |  |  | 0.19 | 0.08 | 0.05 | 0.08 | 0.22 | 0.07 | 0.05 | 0.10 |
|  |  |  |  |  | (0.39) | (0.27) | (0.23) | (0.28) | (0.42) | (0.26) | (0.21) | (0.30) |
| Illness of other household member |  |  |  |  | 0.20 | 0.10 | 0.07 | 0.13 | 0.26 | . | 0.06 | 0.12 |
|  |  |  |  |  | (0.40) | (0.30) | (0.26) | (0.33) | (0.44) | (.) | (0.24) | (0.33) |
| Death or reduction in household members | 0.07 | 0.03 | 0.01 | 0.02 |  |  |  |  |  |  |  |  |
|  | (0.25) | (0.17) | (0.10) | (0.14) |  |  |  |  |  |  |  |  |
| Death of index child's father |  |  |  |  | 0.03 | 0.01 | 0.00 | 0.01 | 0.02 | 0.02 | 0.00 | 0.01 |
|  |  |  |  |  | (0.17) | (0.12) | (0.07) | (0.08) | (0.15) | (0.14) | (0.06) | (0.09) |
| Death of index child's mother |  |  |  |  | 0.02 | 0.00 | 0.00 | 0.01 | 0.02 | 0.01 | 0.00 | 0.00 |
|  |  |  |  |  | (0.14) | (0.07) | (0.04) | (0.08) | (0.12) | (0.09) | (0.05) | (0.07) |
| Death of other household member |  |  |  |  | 0.06 | 0.07 | 0.03 | 0.03 | 0.05 | 0.05 | 0.02 | 0.03 |
|  |  |  |  |  | (0.24) | (0.25) | (0.16) | (0.17) | (0.23) | (0.22) | (0.14) | (0.16) |
| Divorce or separation |  |  |  |  | 0.04 | 0.03 | 0.04 | 0.02 | 0.03 | 0.01 | 0.03 | 0.02 |
|  |  |  |  |  | (0.20) | (0.18) | (0.19) | (0.15) | (0.18) | (0.11) | (0.17) | (0.15) |
| Birth of new household member | 0.10 | 0.08 | 0.08 | 0.08 | 0.23 | 0.19 | 0.12 | 0.18 | 0.17 | 0.05 | 0.11 | 0.08 |
|  | (0.30) | (0.27) | (0.27) | (0.27) | (0.42) | (0.39) | (0.32) | (0.38) | (0.37) | (0.21) | (0.31) | (0.27) |
| Education or school enrolment of child | 0.08 | 0.03 | 0.00 | 0.00 | 0.09 | 0.04 | 0.03 | 0.10 | 0.14 | 0.08 | 0.01 | 0.13 |
|  | (0.27) | (0.18) | (0.06) | (0.07) | (0.29) | (0.20) | (0.17) | (0.30) | (0.35) | (0.27) | (0.10) | (0.34) |
| Number of observations | 1782 | 1840 | 1814 | 1849 | 1782 | 1840 | 1814 | 1849 | 1782 | 1840 | 1814 | 1849 |

*Notes*: Statistics are means with standard deviations in parentheses.

**Table A.4: Descriptive Statistics of Community Prices of Food, Medication, Education and Other Consumption Items by Country**

|  | 2002 | | | | 2006 | | | | 2009 | | | |
| --- | --- | --- | --- | --- | --- | --- | --- | --- | --- | --- | --- | --- |
|  | Ethiopia | India | Peru | Vietnam | Ethiopia | India | Peru | Vietnam | Ethiopia | India | Peru | Vietnam |
| *Food* |  |  |  |  |  |  |  |  |  |  |  |  |
| Cereals | 1.68 |  |  |  | 2.45 |  |  |  | 6.70 |  |  |  |
|  | (0.39) |  |  |  | (0.86) |  |  |  | (0.94) |  |  |  |
| Rice |  | 9.82 | 1.48 | 3.09 |  | 11.80 | 1.70 | 5.09 |  | 20.39 | 1.94 | 8.43 |
|  |  | (2.55) | (0.30) | (0.35) |  | (2.30) | (0.27) | (0.55) |  | (5.31) | (0.45) | (1.81) |
| Potatoes |  |  | 0.63 |  |  |  | 0.85 |  |  |  | 1.02 |  |
|  |  |  | (0.28) |  |  |  | (0.16) |  |  |  | (0.20) |  |
| Pasta |  |  | 2.61 |  |  |  | 2.67 |  |  |  | 3.17 |  |
|  |  |  | (0.46) |  |  |  | (0.39) |  |  |  | (0.34) |  |
| Coffee | 6.86 | 86.70 | 6.75 | 40.16 | 14.24 | 117.28 | 9.41 | 15.89 | 37.80 | 205.71 | 11.86 | 58.70 |
|  | (1.19) | (44.92) | (4.38) | (6.67) | (8.04) | (43.84) | (3.30) | (18.04) | (9.68) | (73.57) | (3.75) | (22.26) |
| Sugar | 4.94 | 15.24 | 1.74 | 6.09 | 8.28 | 19.78 | 2.04 | 9.44 | 14.37 | 34.03 | 2.04 | 15.34 |
|  | (0.38) | (0.71) | (0.31) | (0.62) | (0.81) | (1.59) | (0.19) | (1.27) | (0.63) | (2.66) | (0.24) | (1.05) |
| Oil | 10.76 | 45.89 | 3.75 | 13.96 | 8.88 | 53.78 | 3.97 | 17.60 | 20.03 | 56.52 | 5.87 | 26.29 |
|  | (1.62) | (3.81) | (0.50) | (1.13) | (5.80) | (4.41) | (0.47) | (1.72) | (2.89) | (9.45) | (0.68) | (3.93) |
| Salt | 1.49 | 4.59 | 0.79 | 1.07 | 1.75 | 5.78 | 0.60 | 1.46 | 3.06 | 8.10 | 0.79 | 4.61 |
|  | (0.51) | (1.46) | (0.78) | (0.24) | (2.77) | (1.84) | (0.18) | (0.54) | (0.53) | (2.04) | (0.20) | (2.76) |
| *Medication* |  |  |  |  |  |  |  |  |  |  |  |  |
| Oral rehydration salts | 1.24 | 11.09 | 1.01 | 0.91 | 1.65 | 8.42 | 1.02 | 1.08 | 1.78 | 12.95 | 0.84 | 1.35 |
|  | (0.25) | (5.58) | (0.41) | (0.17) | (0.78) | (4.05) | (0.32) | (0.25) | (0.41) | (7.63) | (0.22) | (0.32) |
| Paracetamol | 0.10 | 0.53 | 0.00 | 0.10 | 0.73 | 0.99 | 0.15 | 0.94 | 0.21 | 1.19 | 0.13 | 2.14 |
|  | (0.02) | (0.17) | (0.00) | (0.06) | (0.60) | (0.94) | (0.07) | (0.59) | (0.16) | (1.27) | (0.06) | (1.35) |
| Amoxicillin | 0.83 | 3.69 | 0.01 | 0.25 | 1.16 | 3.48 | 0.27 | 2.45 | 0.52 | 3.66 | 0.29 | 1.66 |
|  | (0.26) | (1.03) | (0.01) | (0.20) | (0.89) | (0.90) | (0.13) | (1.50) | (0.24) | (0.82) | (0.14) | (1.51) |
| Mebendazole deworming tablets | 2.43 | 4.27 | 0.30 | 0.58 | 1.47 | 7.21 | 0.35 | 2.00 | 0.19 | 13.04 | 0.16 | 3.36 |
|  | (0.11) | (3.91) | (0.27) | (0.45) | (1.57) | (4.79) | (0.22) | (1.85) | (0.07) | (3.55) | (0.10) | (1.79) |
| *Education* |  |  |  |  |  |  |  |  |  |  |  |  |
| Notebook | 2.01 | 4.47 | 1.04 | 0.94 | 2.56 | 5.37 | 1.31 | 2.45 | 3.36 | 6.15 | 1.43 | 4.18 |
|  | (0.85) | (1.06) | (0.21) | (0.18) | (0.41) | (1.40) | (0.20) | (0.80) | (0.91) | (2.59) | (0.18) | (1.97) |
| School shoes | 25.03 | 110.65 | 28.87 | 14.99 | 29.86 | 110.69 | 30.90 | 32.69 | 56.58 | 128.92 | 37.14 | 25.37 |
|  | (17.85) | (78.64) | (4.29) | (6.00) | (20.71) | (36.92) | (3.76) | (19.53) | (28.44) | (33.75) | (6.86) | (15.28) |
| Boy's shirt | 19.31 | 94.55 | 12.80 | 14.78 | 21.03 | 68.35 | 10.93 | 26.35 | 30.57 | 112.65 | 13.75 | 30.78 |
|  | (10.37) | (32.61) | (3.37) | (3.70) | (9.10) | (32.36) | (2.72) | (7.15) | (15.24) | (48.15) | (2.74) | (8.07) |
| Girl's shirt | 17.13 | 104.80 | 12.63 | 15.34 | 25.49 | 74.43 | 10.63 | 25.48 | 38.18 | 109.97 | 13.66 | 32.23 |
|  | (8.70) | (54.35) | (3.00) | (4.06) | (10.11) | (45.93) | (2.88) | (6.70) | (17.61) | (56.22) | (2.67) | (8.42) |
| Boy's shorts | 19.65 | 102.14 | 20.48 | 9.15 | 26.10 | 101.35 | 24.16 | 9.95 | 52.37 | 183.28 | 27.22 | 15.72 |
|  | (12.60) | (69.58) | (5.39) | (1.41) | (18.77) | (42.62) | (4.47) | (4.39) | (46.55) | (70.13) | (3.90) | (6.83) |
| Girl's skirt | 19.76 | 110.62 | 18.13 | 17.21 | 29.62 | 91.89 | 20.78 | 28.42 | 53.43 | 163.06 | 23.96 | 30.38 |
|  | (9.57) | (59.59) | (4.87) | (6.74) | (14.78) | (30.36) | (3.13) | (11.31) | (47.92) | (75.75) | (3.59) | (7.77) |
| *Other consumption items* |  |  |  |  |  |  |  |  |  |  |  |  |
| Cigarettes | 3.91 | 29.35 | 3.19 | 1.99 | 1.77 | 18.28 | 3.81 | 6.44 | 5.78 | 24.08 | 3.80 | 10.31 |
|  | (0.65) | (14.81) | (0.87) | (0.03) | (1.77) | (2.71) | (0.89) | (3.10) | (0.75) | (7.24) | (1.06) | (3.86) |
| Detergent | 4.65 | 14.30 | 2.07 | 13.05 | 1.63 | 6.23 | 1.62 | 17.53 | 23.79 | 8.20 | 1.18 | 25.76 |
|  | (4.91) | (7.39) | (1.03) | (1.48) | (1.02) | (2.96) | (0.43) | (2.89) | (8.07) | (4.32) | (0.19) | (2.89) |
| Kerosene | 2.53 | 14.61 | 1.85 | 4.33 | 4.07 | 16.49 | 12.61 | 10.20 | 8.96 | 12.63 | 14.29 | 15.78 |
|  | (0.75) | (2.02) | (0.35) | (0.44) | (1.32) | (4.82) | (2.07) | (3.26) | (0.92) | (4.19) | (1.83) | (1.42) |
| Number of observations | 1782 | 1840 | 1814 | 1849 | 1782 | 1840 | 1814 | 1849 | 1782 | 1840 | 1814 | 1849 |

*Notes*: Statistics are means with standard deviations in parentheses. Prices are in national currency units. Prices of food items are per kg except for oil for which price is per lt. Price of cereals in Ethiopia is the average of the prices of white teff, sorghum, and barley. Prices of medication are per tablet except for oral rehydration salts for which price is per sachet. Price for cigarettes is for one pack of 20 and price for kerosene is per lt. Prices were combined to calculate Paasche price indices for food, medication, education, and other consumption items using equal weights except for the food price index for which weights used were the share of each item in the total consumption expenditure in the community computed using information on household consumption expenditure. Base prices in the price index were the median prices of the items used.

**Table A.5: Descriptive Statistics of Community Characteristics by Country**

|  | 2002 | | | | 2006 | | | | 2009 | | | |
| --- | --- | --- | --- | --- | --- | --- | --- | --- | --- | --- | --- | --- |
|  | Ethiopia | India | Peru | Vietnam | Ethiopia | India | Peru | Vietnam | Ethiopia | India | Peru | Vietnam |
| Number of credit-providing institutions in community | 2.02 | 2.52 | 1.55 | 2.69 |  |  |  |  |  |  |  |  |
|  | (1.07) | (0.92) | (1.21) | (1.12) |  |  |  |  |  |  |  |  |
| Average wage of adult male agricultural worker | 5.41 | 45.74 | 11.45 | 20.30 | 9.98 | 61.07 | 12.61 | 35.27 | 19.26 | 120.01 | 17.33 | 73.20 |
|  | (1.85) | (10.21) | (3.30) | (4.94) | (2.23) | (11.27) | (4.05) | (5.92) | (5.03) | (29.85) | (4.50) | (15.81) |
| Average wage of adult male unskilled factory worker | 8.17 | 50.01 | 21.57 | 22.64 | 8.57 | 70.56 | 15.76 | 27.81 | 507.41 | 135.63 | 22.99 | 57.66 |
|  | (2.08) | (2.98) | (1.25) | (4.83) | (1.52) | (14.16) | (3.80) | (7.73) | (131.01) | (23.38) | (3.13) | (10.52) |
| Air pollution is a severe problem in community | 0.79 | 0.90 | 0.48 | 0.55 | 0.63 | 0.58 | 0.37 | 0.16 | 0.56 | 0.57 | 0.41 | 0.15 |
|  | (0.41) | (0.30) | (0.50) | (0.50) | (0.48) | (0.49) | (0.48) | (0.37) | (0.50) | (0.50) | (0.49) | (0.36) |
| Water pollution is a severe problem in community | 0.48 | 0.80 | 0.40 | 0.38 | 0.60 | 0.83 | 0.53 | 0.45 | 0.49 | 0.72 | 0.62 | 0.22 |
|  | (0.50) | (0.40) | (0.49) | (0.48) | (0.49) | (0.38) | (0.50) | (0.50) | (0.50) | (0.45) | (0.49) | (0.42) |
| Improved water available in community | 0.69 | 0.94 | 0.99 | 0.70 | 0.95 | 0.98 | 0.94 | 0.82 | 0.81 | 0.79 | 0.96 | 0.98 |
|  | (0.46) | (0.24) | (0.09) | (0.46) | (0.22) | (0.13) | (0.24) | (0.38) | (0.39) | (0.41) | (0.20) | (0.15) |
| Improved toilet facilities available in community | 0.65 | 0.63 | 0.94 | 0.78 | 1.00 | 0.92 | 0.96 | 1.00 | 0.84 | 0.98 | 0.95 | 0.96 |
|  | (0.48) | (0.48) | (0.24) | (0.41) | (0.00) | (0.27) | (0.19) | (0.00) | (0.36) | (0.14) | (0.21) | (0.19) |
| Garbage disposal truck available in community | 0.13 | 0.19 | 0.51 | 0.26 | 0.20 | 0.28 | 0.67 | 0.36 | 0.10 | 0.27 | 0.69 | 0.53 |
|  | (0.33) | (0.39) | (0.50) | (0.44) | (0.40) | (0.45) | (0.47) | (0.48) | (0.29) | (0.45) | (0.46) | (0.50) |
| Hospital present in community | 0.64 | 0.38 | 0.52 | 0.98 | 0.49 | 0.42 | 0.58 | 0.98 | 0.60 | 0.36 | 0.55 | 0.88 |
|  | (0.48) | (0.49) | (0.50) | (0.13) | (0.50) | (0.49) | (0.49) | (0.14) | (0.49) | (0.48) | (0.50) | (0.32) |
| Number of schools in community |  |  |  |  | 0.92 | 2.60 | 2.19 | 1.37 | 5.60 | 7.27 | 1.16 | 6.19 |
|  |  |  |  |  | (0.96) | (1.76) | (0.80) | (0.70) | (1.55) | (3.29) | (0.69) | (3.34) |
| Number of observations | 1782 | 1840 | 1814 | 1849 | 1782 | 1840 | 1814 | 1849 | 1782 | 1840 | 1814 | 1849 |

*Notes*: Statistics are means with standard deviations in parentheses. Wages are in national currency units. A wage index was constructed by dividing the average wage of adult male agricultural worker in rural communities and the average wage for adult male unskilled factory worker in urban communities with their median analogue. Access to improved drinking water and sanitation here means that, during data collection, the household had access to improved drinking water and toilets as defined by WHO/UNICEF (see <http://www.wssinfo.org/definitions-methods/watsan-categories/>), not that access to drinking water and sanitation improved between rounds of data collection.

**Table A.6: OLS Estimates of Associations of Child Growth Trajectories from Age 1 to 8 Years with Achievement Scores at Ages 8 and 12 Years**

|  | Ethiopia | | | | India | | | | Peru | | | | Vietnam | | | |
| --- | --- | --- | --- | --- | --- | --- | --- | --- | --- | --- | --- | --- | --- | --- | --- | --- |
|  | PPVT at 8 y | Maths at 8 y | PPVT at 12 y | Maths at 12 y | PPVT at 8 y | Maths at 8 y | PPVT at 12 y | Maths at 12 y | PPVT at 8 y | Maths at 8 y | PPVT at 12 y | Maths at 12 y | PPVT at 8 y | Maths at 8 y | PPVT at 12 y | Maths at 12 y |
| SSS: Stunted at ages 1, 5, and 8 y | -0.19*** | -0.30*** | -0.23*** | -0.29*** | -0.31*** | -0.42*** | -0.36*** | -0.23*** | -0.23*** | -0.32*** | -0.32*** | -0.23*** | -0.12* | -0.34*** | -0.25*** | -0.17** |
|  | (0.06) | (0.07) | (0.07) | (0.07) | (0.06) | (0.06) | (0.07) | (0.06) | (0.07) | (0.07) | (0.06) | (0.07) | (0.07) | (0.07) | (0.08) | (0.07) |
| SSN: Stunted at ages 1 and 5 y | -0.13 | -0.16* | -0.07 | -0.13 | -0.24** | -0.26** | -0.31*** | -0.17 | -0.03 | -0.11 | -0.30*** | -0.12 | -0.14 | -0.16 | -0.15 | -0.14 |
|  | (0.08) | (0.09) | (0.08) | (0.09) | (0.09) | (0.11) | (0.11) | (0.11) | (0.10) | (0.10) | (0.10) | (0.10) | (0.08) | (0.10) | (0.13) | (0.10) |
| SNS: Stunted at ages 1 and 8 y | -0.41*** | -0.23 | -0.48** | -0.10 | -0.35*** | -0.47*** | -0.19 | -0.28 | -0.57*** | -0.63*** | -0.48*** | -0.52** | -0.36* | -0.55** | -0.46 | -0.39** |
|  | (0.12) | (0.16) | (0.19) | (0.17) | (0.13) | (0.15) | (0.16) | (0.18) | (0.20) | (0.23) | (0.17) | (0.26) | (0.19) | (0.26) | (0.31) | (0.16) |
| SNN: Stunted at age 1 y | -0.11** | -0.16*** | -0.11** | -0.12* | -0.21*** | -0.21*** | -0.26*** | -0.19** | -0.15** | -0.13* | -0.14** | -0.11 | -0.02 | -0.15 | 0.02 | -0.11 |
|  | (0.05) | (0.06) | (0.05) | (0.06) | (0.08) | (0.07) | (0.07) | (0.07) | (0.07) | (0.08) | (0.07) | (0.08) | (0.10) | (0.09) | (0.10) | (0.10) |
| NSS: Stunted at ages 5 and 8 y | -0.26** | -0.41*** | -0.41*** | -0.25* | -0.16** | -0.03 | -0.15** | -0.12 | -0.29*** | -0.26** | -0.36*** | -0.25** | -0.10 | 0.17** | -0.09 | -0.06 |
|  | (0.11) | (0.11) | (0.11) | (0.14) | (0.07) | (0.08) | (0.08) | (0.08) | (0.09) | (0.10) | (0.09) | (0.11) | (0.10) | (0.08) | (0.10) | (0.09) |
| NSN: Stunted at age 5 y | -0.03 | -0.08 | -0.02 | -0.05 | 0.18* | 0.02 | 0.00 | -0.06 | -0.10 | -0.09 | -0.23*** | -0.14* | 0.01 | 0.05 | -0.10 | 0.15 |
|  | (0.09) | (0.08) | (0.08) | (0.09) | (0.11) | (0.10) | (0.08) | (0.08) | (0.06) | (0.07) | (0.06) | (0.08) | (0.09) | (0.09) | (0.11) | (0.11) |
| NNS: Stunted at age 8 y | -0.15 | -0.23 | -0.29* | -0.29* | 0.07 | 0.21 | -0.05 | 0.07 | -0.23** | -0.07 | -0.33*** | -0.13 | -0.28*** | 0.14 | -0.18 | -0.05 |
|  | (0.14) | (0.16) | (0.16) | (0.16) | (0.14) | (0.15) | (0.11) | (0.14) | (0.11) | (0.15) | (0.13) | (0.14) | (0.11) | (0.13) | (0.14) | (0.13) |
| Male | -0.01 | 0.04 | 0.06 | 0.00 | 0.24*** | 0.08* | 0.14*** | 0.02 | 0.05 | 0.11*** | 0.17*** | 0.05 | 0.05 | -0.00 | 0.02 | -0.08* |
|  | (0.04) | (0.04) | (0.04) | (0.05) | (0.04) | (0.04) | (0.04) | (0.04) | (0.04) | (0.04) | (0.04) | (0.04) | (0.04) | (0.04) | (0.04) | (0.04) |
| Second-born | -0.02 | 0.06 | -0.02 | 0.07 | -0.05 | -0.01 | -0.06 | -0.04 | 0.02 | -0.06 | 0.06 | 0.04 | -0.09* | -0.07 | -0.13*** | -0.01 |
|  | (0.07) | (0.06) | (0.06) | (0.07) | (0.05) | (0.05) | (0.05) | (0.05) | (0.04) | (0.05) | (0.05) | (0.05) | (0.05) | (0.04) | (0.04) | (0.05) |
| Third- or later-born | 0.03 | -0.01 | -0.08 | -0.03 | -0.20*** | -0.19*** | -0.23*** | -0.23*** | 0.02 | -0.16*** | -0.02 | 0.01 | -0.17*** | -0.06 | -0.34*** | -0.16*** |
|  | (0.06) | (0.05) | (0.05) | (0.06) | (0.06) | (0.06) | (0.07) | (0.06) | (0.05) | (0.06) | (0.05) | (0.06) | (0.06) | (0.06) | (0.07) | (0.06) |
| Child age in 2002 | -0.01** | -0.01 | -0.01** | -0.00 | 0.00 | 0.00 | 0.00 | -0.01 | 0.01 | 0.01 | 0.01*** | 0.01 | -0.02*** | -0.01* | -0.01** | -0.02*** |
|  | (0.01) | (0.01) | (0.01) | (0.01) | (0.01) | (0.01) | (0.01) | (0.01) | (0.01) | (0.01) | (0.01) | (0.01) | (0.01) | (0.01) | (0.01) | (0.01) |
| Months between interviews in 2002 and 2006 | -0.04* | 0.03 | -0.03 | 0.04 | -0.06*** | -0.03 | -0.02 | -0.02 | 0.04** | 0.02 | 0.02 | -0.02 | 0.03* | -0.01 | -0.02 | 0.05*** |
|  | (0.02) | (0.02) | (0.02) | (0.03) | (0.02) | (0.02) | (0.02) | (0.02) | (0.02) | (0.03) | (0.02) | (0.03) | (0.02) | (0.02) | (0.02) | (0.02) |
| Months between interviews in 2006 and 2009 | 0.02 | 0.06** | 0.00 | 0.04 | 0.00 | 0.06*** | 0.03* | 0.04** | 0.01 | 0.03 | -0.02 | -0.03 | -0.02 | -0.00 | 0.07** | 0.08*** |
|  | (0.03) | (0.03) | (0.03) | (0.03) | (0.02) | (0.02) | (0.02) | (0.02) | (0.02) | (0.03) | (0.02) | (0.03) | (0.02) | (0.02) | (0.03) | (0.02) |
| Caregiver's age at index child’s birth | 0.01** | 0.01** | 0.00** | 0.00 | 0.01** | 0.01 | 0.00 | 0.00 | 0.00 | 0.00 | 0.00* | 0.00 | 0.01*** | 0.00 | 0.00 | 0.00 |
|  | (0.00) | (0.00) | (0.00) | (0.00) | (0.00) | (0.01) | (0.00) | (0.00) | (0.00) | (0.00) | (0.00) | (0.00) | (0.00) | (0.00) | (0.00) | (0.00) |
| Caregiver's schooling | 0.05*** | 0.06*** | 0.04*** | 0.04*** | 0.03*** | 0.04*** | 0.03*** | 0.05*** | 0.04*** | 0.04*** | 0.06*** | 0.06*** | 0.06*** | 0.03*** | 0.03*** | 0.04*** |
|  | (0.01) | (0.01) | (0.01) | (0.01) | (0.01) | (0.01) | (0.01) | (0.01) | (0.01) | (0.01) | (0.01) | (0.01) | (0.01) | (0.01) | (0.01) | (0.01) |
| Father's schooling | 0.04*** | 0.05*** | 0.05*** | 0.05*** | 0.03*** | 0.02*** | 0.03*** | 0.04*** | 0.05*** | 0.03*** | 0.04*** | 0.03*** | 0.03*** | 0.04*** | 0.02*** | 0.05*** |
|  | (0.01) | (0.01) | (0.01) | (0.01) | (0.01) | (0.01) | (0.01) | (0.01) | (0.01) | (0.01) | (0.01) | (0.01) | (0.01) | (0.01) | (0.01) | (0.01) |
| Log household expenditure in 2009 less spending on index child health in 2006 and 2009 | 0.24*** | 0.17*** | 0.20*** | 0.12*** | 0.07 | 0.04 | -0.02 | -0.02 | 0.22*** | 0.24*** | 0.16*** | 0.14*** | 0.08 | 0.17*** | 0.21*** | 0.09** |
|  | (0.04) | (0.05) | (0.04) | (0.04) | (0.05) | (0.04) | (0.04) | (0.05) | (0.04) | (0.04) | (0.04) | (0.04) | (0.05) | (0.04) | (0.04) | (0.05) |
| Number of schools in community in 2009 | -0.06*** | -0.08*** | -0.13*** | -0.09*** | -0.01 | -0.05*** | -0.04*** | -0.04*** | -0.04 | 0.02 | -0.09*** | -0.10*** | 0.00 | 0.05*** | -0.00 | -0.02** |
|  | (0.02) | (0.02) | (0.01) | (0.02) | (0.01) | (0.01) | (0.01) | (0.01) | (0.03) | (0.03) | (0.03) | (0.03) | (0.01) | (0.01) | (0.01) | (0.01) |
| Community wage index in 2009 | 0.61*** | 0.25** | 0.24*** | 0.08 | 0.08 | -0.07 | 0.51*** | -0.01 | 0.64*** | 0.51*** | 0.32*** | 0.18 | -0.38** | 0.61*** | 0.00 | -0.02 |
|  | (0.10) | (0.10) | (0.09) | (0.11) | (0.13) | (0.13) | (0.13) | (0.13) | (0.12) | (0.11) | (0.11) | (0.11) | (0.16) | (0.16) | (0.16) | (0.18) |
| Number of credit-providing institutions in the community in 2002 | -0.05* | -0.01 | -0.02 | 0.11*** | -0.02 | 0.00 | -0.07*** | 0.01 | 0.04*** | -0.01 | 0.04** | 0.04** | -0.04* | 0.07*** | -0.07*** | -0.02 |
|  | (0.03) | (0.03) | (0.02) | (0.03) | (0.03) | (0.02) | (0.02) | (0.02) | (0.01) | (0.02) | (0.02) | (0.02) | (0.02) | (0.02) | (0.02) | (0.02) |
| Consumption price index in 2009 | 0.31*** | -0.42*** | 0.35*** | -0.35*** | -0.19 | -0.15 | 0.09 | 0.01 | 0.30* | 0.21 | 0.38** | 0.67*** | 0.48*** | 1.14*** | -0.03 | -0.53*** |
|  | (0.10) | (0.13) | (0.11) | (0.13) | (0.17) | (0.14) | (0.14) | (0.15) | (0.16) | (0.17) | (0.15) | (0.16) | (0.15) | (0.16) | (0.17) | (0.16) |
| Education price index in 2009 | -0.58*** | -0.29*** | -0.63*** | -0.21** | -0.25*** | -0.24*** | -0.12* | -0.34*** | 0.07 | 0.09 | 0.01 | -0.25 | -0.22 | -0.22* | -0.14 | -0.64*** |
|  | (0.08) | (0.08) | (0.07) | (0.09) | (0.07) | (0.07) | (0.07) | (0.07) | (0.16) | (0.19) | (0.17) | (0.18) | (0.14) | (0.13) | (0.14) | (0.14) |
| Constant | 0.38 | -3.82* | 1.28 | -2.94 | 2.69* | 0.43 | -0.75 | -0.45 | -5.49*** | -4.68** | -3.65** | -0.84 | -1.74 | -2.78** | -2.47* | -4.91*** |
|  | (1.91) | (2.04) | (1.88) | (2.37) | (1.63) | (1.49) | (1.55) | (1.42) | (1.69) | (2.32) | (1.65) | (2.19) | (1.35) | (1.24) | (1.42) | (1.27) |
|  |  |  |  |  |  |  |  |  |  |  |  |  |  |  |  |  |
| R-squared | 0.41 | 0.36 | 0.41 | 0.28 | 0.16 | 0.22 | 0.23 | 0.25 | 0.44 | 0.31 | 0.40 | 0.26 | 0.27 | 0.29 | 0.28 | 0.23 |
| Observations | 1,540 | 1,501 | 1,540 | 1,501 | 1,813 | 1,784 | 1,813 | 1,784 | 1,715 | 1,756 | 1,715 | 1,756 | 1,737 | 1,778 | 1,737 | 1,778 |

*Notes*: Robust standard errors in parentheses. *** significant at 1%, ** significant at 5%, * significant at 10%. Dependent variables are age-normalised test scores. Regressions include controls for caregiver’s ethnicity, language of administration of the tests, and whether the tests were administered in the child’s native tongue, but estimates are not reported.

**Appendix References**

Cameron, N., Preece, M. A., & Cole, T. J. (2005). Catch-up Growth or Regression to the Mean? Recovery from Stunting Revisited. *American Journal of Human Biology*, *17*(4), 412–417. https://doi.org/10.1002/ajhb.20408

Eckhardt, C. l., Gordon-Larsen, P., & Adair, L. S. (2005). Growth patterns of Filipino children indicate potential compensatory growth. *Annals of Human Biology*, *32*(1), 3–14. https://doi.org/10.1080/03014460400027607

Godoy, R., Nyberg, C., Eisenberg, D. T. A., Magvanjav, O., Shinnar, E., Leonard, W. R., … Tanner, S. (2010). Short but catching up: Statural growth among native Amazonian Bolivian children. *American Journal of Human Biology*, *22*(3), 336–347. https://doi.org/10.1002/ajhb.20996
